# Design, Assessment, and Validation of a Questionnaire to Estimate Food-Dependent Exercise-Induced Anaphylaxis Prevalence in Latin American Population

**DOI:** 10.3390/healthcare8040519

**Published:** 2020-11-29

**Authors:** Jhonatan González-Santamaría, Jesús Gilberto Arámburo-Gálvez, Carlos Eduardo Beltrán-Cárdenas, José Antonio Mora-Melgem, Oscar Gerardo Figueroa-Salcido, Giovanni Isaí Ramírez-Torres, Feliznando Isidro Cárdenas-Torres, Itallo Carvalho Gomes, Tatiane Geralda André, María Auxiliadora Macêdo-Callou, Élida Mara Braga Rocha, Noé Ontiveros, Francisco Cabrera-Chávez

**Affiliations:** 1 Nutrition Sciences, Faculty of Nutrition Sciences, University of Sinaloa, Culiacán 80019, Sinaloa, Mexico; jgonzalez@utp.edu.co (J.G.-S.); carlos.1.beltran@hotmail.com (C.E.B.-C.); joseantoniomoramelgem@gmail.com (J.A.M.-M.); giovannirt2@hotmail.com (G.I.R.-T.); feliznando@uas.edu.mx (F.I.C.-T.); 2 Faculty of Health and Sports Sciences, University Foundation of the Andean Area, Pereira 66001, Risaralda, Colombia; 3 Health Sciences, Division of Biological and Health Sciences, University of Sonora, Hermosillo 83000, Sonora, Mexico; gilberto.aramburo.g@gmail.com (J.G.A.-G.); gerardofs95@hotmail.com (O.G.F.-S.); 4 Science Program in Nursing, School of Nursing, Los Mochis 81220, Sinaloa, Mexico; carvalhoitallo@gmail.com (I.C.G.); tatianegrandre@gmail.com (T.G.A.); 5 Department of Nutrition, University Center of Juazeiro do Norte, Juazeiro do Norte, Juazeiro do Norte 63010-215, Ceara, Brazil; auxiliadora.callou@unijuazeiro.edu.br (M.A.M.-C.); elida.braga@unijuazeiro.edu.br (É.M.B.R.); 6 Clinical and Research Laboratory (LACIUS, URS), Department of Chemical, Biological, and Agricultural Sciences (DC-QB), Division of Sciences and Engineering, University of Sonora, Navojoa 85880, Sonora, Mexico

**Keywords:** food-dependent exercise-induced anaphylaxis, survey studies, questionnaire design

## Abstract

There are no epidemiological data about food-dependent exercise-induced anaphylaxis (FDEIA) in Latin America. Our aim was to design, assess, and validate a questionnaire to identify potential FDEIA cases and/or estimate its prevalence by self-report. Questions were included in the instrument to address the main symptoms of FDEIA, type/intensity of physical activity, and anaphylaxis. The instrument’s clarity, comprehension and repeatability were evaluated. These evaluations were carried out by Hispanic people (Argentinians/Colombians/Mexicans/Peruvians), including nine individuals with medical diagnosis of FDEIA, and Brazilians. The Flesch–Kincaid score was calculated using the INFLESZ software. The instrument was translated from Spanish to Brazilian Portuguese following the translation back-translation procedure. The participants rated the two versions of the questionnaire as clear and comprehensible (three-point ordinal scale) and very easy to understand [0.33; average (scale 0–10)]. For these evaluations, the Kendall’s W coefficient showed strong agreement among raters (W = 0.80; average). The Flesch–Kincaid score was 63.5 in average (documents considered as readable). The Cohen’s Kappa coefficient showed almost perfect agreement in repeatability (0.88; average). The validation process of two versions of an instrument, used to identify potential FDEIA cases, was successfully carried out and it was found applicable to Latin American countries for generating epidemiological data.

## 1. Introduction

Food-dependent exercise-induced anaphylaxis (FDEIA) is a disorder, characterized by the development of anaphylactic reactions, occurring after eating specific allergenic foods and performing exercise [1]. Only a few epidemiological studies have estimated the prevalence of FDEIA [2,3]. These studies were carried out in Japan and reported prevalence rates of 0%, 0.06%, and 0.017–0.21% among children from kindergartens, primary school students, and junior high school students, respectively. Due to FDEIA is rare, life-threatening, and commonly underdiagnosed [4], awareness about this condition should be increased among medical personnel [5]. Symptoms commonly reported in FDEIA cases, include angioedema, dyspnea, pruritus, repetitive cough, wheezing, gastrointestinal symptoms, fatigue, loss of consciousness, and hypotension [4,6]. These symptoms have been the hallmark of the condition since the first FDEIA case was reported in 1979 [7,8,9].

The diagnosis of FDEIA involves a food challenge with the suspected food to rule out food-induced anaphylaxis. This procedure is followed by a food challenge in combination with exercise to corroborate that the anaphylactic symptoms reported are exercise-dependent ones [10]. A double-blind placebo-controlled food challenge with exercise is recommended in cases where the results are difficult to interpret. These interventions must be carried out by highly trained healthcare personnel, under special clinical setting, and after the patient has received proper diagnostic counseling for minimizing the stress triggered for the possibility of developing anaphylactic symptoms. These reasons make it difficult and expensive to estimate, at a population level, the gold standard-based prevalence of FDEIA. Alternatively, survey studies, using validated questionnaires for obtaining data about self-reported adverse food reactions, triggered while, or after, performing exercise are useful for identifying potential FDEIA cases and generating epidemiological data. Notably, anaphylaxis is a well characterized reaction, which is relatively easy to identify by self-report of symptoms [11,12,13]. However, there is neither Spanish nor Portuguese version of a validated questionnaire to identify potential FDEIA cases or to generate epidemiological data. Certainly, survey tools have to be designed taking into account the scientific knowledge [14] and their clarity, comprehension and consistency be assessed systematically for ensuring the repeatability of the results [15,16,17]. Therefore, our aim was to design, assess and validate a questionnaire to estimate the prevalence of FDEIA in both Spanish and Brazilian Portuguese speaking populations as these two languages are predominant in the Latin American region.

## 2. Materials and Methods

### 2.1. Questionnaire Design

**Figure 1:**
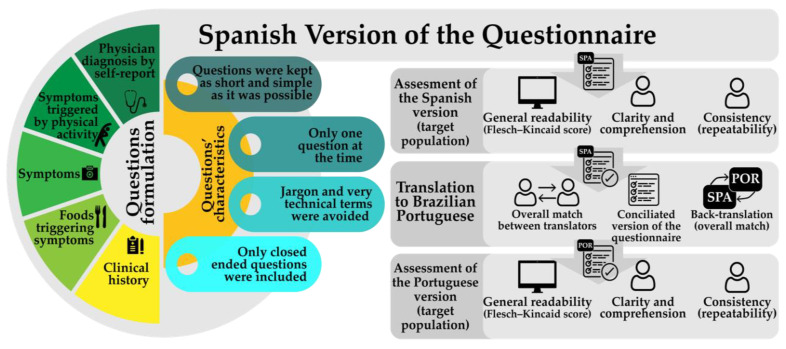
Scheme followed to design and evaluate the questionnaire.

Figure 1 shows the scheme followed to design and evaluate the questionnaire. The items of the questionnaire were designed based on the FDEIA symptoms most frequently reported in scientific literature [3,18,19,20] and published instruments intended to detect cases of anaphylaxis [11,12,13]. The questionnaire collect data about clinical history, symptoms triggered after food ingestion, while or after performing exercise, or after a combination of both food ingestion and exercise, and the level of exercise intensity, among others. The level of exercise intensity was based on the capability to realize activities such as speaking, singing, and/or whistle [21]. The questions were kept as short and simple as possible, and jargon and technical terms were avoided.

### 2.2. Assessment of Clarity and Comprehension of the Spanish Version of the Questionnaire

The questionnaire was digitalized using the SurveyMonkey platform (San Mateo, CA, USA). The hyperlink that was generated to evaluate the instrument was sent via text message to Spanish native speakers from México, Colombia, Argentina, and Peru. Clarity and comprehension of the questionnaire were assessed, as previously described [17]. Briefly: For clarity assessment of the questionnaire a three-point ordinal scale (3: Clear and comprehensible, 2: Difficult to understand, and 1: Incomprehensible) was used. For comprehension assessment, a numerical scale from 0 to 10 (0 = very easy to understand; 10 = very difficult to understand) was used. Questions with values of 1 or ≥2 (three-point ordinal scale) without suggestions of rewording and rated ≤3 (numerical scale from 0 to 10) for >90% of the participants were considered comprehensible and rewording was not necessary. Additionally, clarity/comprehension was evaluated using the Flesch–Kincaid readability tests for Spanish text [22], which has also been successfully applied for Portuguese texts [23]. The Flesch–Kincaid score ranges from 0 to 100. A score of 0 means that the text is very complicated to read while a score of 100 means that the text is very easy to read. The Flesch–Kincaid score was calculated using the INFLESZ software (Granada, Granada, Spain) [24]. A score ≥60 was considered as readable [22]. Agreement among raters was assessed employing the Kendall’s W coefficient of concordance, ranging from 0 (no agreement) to 1 (complete agreement). A W value ≥0.70 was considered as adequate agreement among raters [17].

### 2.3. Assessment of Consistency (Repeatability)

The questionnaire was consistency evaluated by test-retest analysis in a cohort of healthy subjects, and in a cohort of FDEIA subjects who reported that a physician diagnosed them. Subjects from four Hispanic countries (México, Colombia, Argentina, and Peru) participated at this stage of the study. The participants answered the questionnaire twice with at least one-week interval between the first and second application. The repeatability of the questionnaire was evaluated with the Cohen’s kappa coefficient test. At this stage of the evaluations, the time to answer the questionnaire for the first time was recorded.

### 2.4. Questionnaire Translation to Portuguese Back-Translation Procedure

The final Spanish version of the questionnaire was translated to Portuguese by two native Portuguese speakers (bilinguals Portuguese/Spanish) and a conciliated version of it was back translated to Spanish to ensure that the meaning of the questions was not loss. This procedure was carried out following previously described methodologies [17]. Briefly, two bilingual Portuguese-Spanish health professionals (native Portuguese speakers) translated the questionnaire from Spanish to Portuguese. Overall matches (expressed as percentages) between the translated versions of the questionnaire were determined by the WcopyFind software 4.1.5 (Charlottesville, VA, USA) ignoring all punctuation marks, numbers, uppercase and lowercase. Brazilian-Portuguese was selected as the base language in the software. A conciliation of the conflicting words (words with no coincidences) was agreed by two Spanish-Portuguese translators. The conciliated Portuguese version of the questionnaire was back translated from Portuguese to Spanish by two bilingual Spanish/Portuguese (native Spanish speakers). The overall match between the original Spanish version of the questionnaire and each back-translated Spanish version of it was evaluated as it was described above but selecting Spanish as the base language. Finally, clarity, comprehension and consistency of the conciliated Portuguese version of the questionnaire were evaluated as described in Section 2.2 and Section 2.3, but the hyperlink to evaluate the instrument was sent via text message to Portuguese native speakers from both Ceará and Mato Grosso do Sul states.

### 2.5. Statistical and Ethical Issues

The coefficients Kendall’s W and Cohen’s kappa were calculated using PASW Statistics Version 25.0 (SPSS Inc., Chicago, IL, USA). The total numbers, percentages, means and standard deviations (SD) were analyzed using descriptive statistics (PASW Statistics Version 25.0, SPSS Inc., Chicago, IL, USA). The study was approved by the ethics committee of the Faculty of Nutrition and Gastronomy of the Autonomous University of Sinaloa (ethical approval number: CE-UACNyG-2020-JUL-001) considering that there is no risk of affecting the standards of the Declaration of Helsinki. The basic principles of Bioethics and the individual and social principles of UNESCO were respected. Participants’ identities were not disclosed. Signed informed consent was not required, but information about the investigators responsible for data handling and about publishing the results was provided. All questions were designed to ensure they do not threaten human dignity or cultural diversity.

## 3. Results

### 3.1. Questionnaire Design

**Figure 2:**
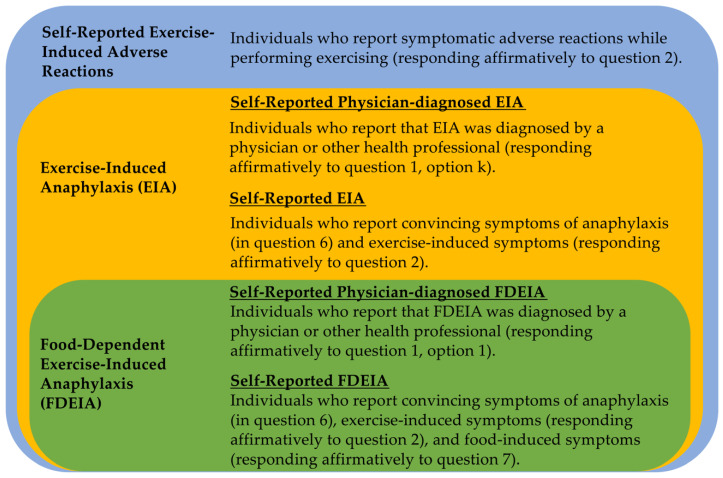
Algorithm for allocating the interviewees into the category of self-reported exercise-induced adverse reactions, self-reported physician diagnosed EIA, self-reported EIA, self-reported physician-diagnosed FDEIA, or self-reported FDEIA.

The questionnaire developed includes nineteen questions covering the main symptoms and situations that can suggest FDEIA (Supplemental Material Table S1). Participants can be allocated into three categories including two subcategories (Figure 2). Participants allocated in the categories exercise induced anaphylaxis (EIA) and FDEIA must meet well stablished criteria of anaphylaxis [25]. Demographic and clinical data, such as age/gender and physician-diagnosed atopic diseases, are also collected. Figure 2 shows an algorithm for making decisions for allocating interviewees who report exercise-induced adverse reactions. For identifying anaphylactic cases by self-report, the interviewees must meet at least one of the following three conditions: (1) Acute onset of an illness with involvement of the skin, mucosal tissue or both and respiratory compromise or reduced blood pressure. (2) Two or more of the following that occur rapidly after food ingestion/exercise: (a) involvement of the skin-mucosal tissue, (b) respiratory compromise, (c) reduced blood pressure. (3) Reduced blood pressure after exposure to a food allergen/exercise [25].

### 3.2. Translation of the Questionnaire to Portuguese/Back-Translation to Spanish

**Figure 3:**
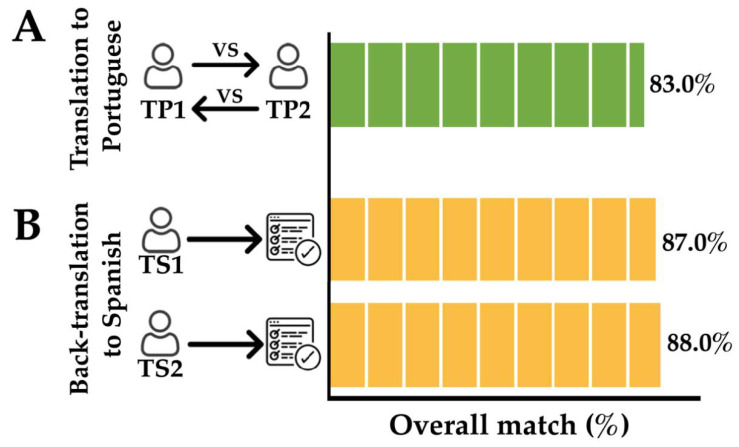
Assessment of questionnaire translation from Spanish to Portuguese and back-translation from Portuguese to Spanish. Part (**A**): TP1 and TP2: translators to Portuguese 1 and 2, respectively (bilingual Portuguese/Spanish; Portuguese native speakers); Part (**B**): TS1 and TS2: back-translators to Spanish 1 and 2, respectively (bilingual Spanish/Portuguese; Spanish native speakers).

The overall match between the translated versions of the questionnaire from Spanish to Portuguese was 83% (procedure carried out by two bilingual Portuguese/Spanish (Portuguese native speakers) (Figure 3A). The items in conflict were synonymous in Portuguese, which have the same meaning in Spanish language. Two Spanish-Portuguese translators selected the most common synonymous in order to generate a conciliated Portuguese version of the questionnaire. The overall matches between the original Spanish version of the questionnaire and the back-translations to Spanish (from the conciliated Portuguese version) were 87% and 88% (Figure 3B). This procedure was carried out by two bilingual Spanish/Portuguese (Spanish native speakers). The items in conflict were synonymous.

### 3.3. Characteristics of the Evaluators of the Spanish and Portuguese Versions of the Questionnaire

The characteristics of participants who evaluated clarity, comprehension and consistency of the questionnaire are shown in Table 1. Age was ranged from 18 to 68 years old (mean 29.2 ± 9.8) and participants had a scholarly from elementary school to post-graduate. Native Spanish or native Portuguese speakers evaluated the clarity, comprehension and consistency of all items/questions of the Spanish or Portuguese version of the questionnaire, respectively. Additionally, a cohort of FDEIA subjects who reported that a physician diagnosed them evaluated the questionnaire. This cohort included individuals from México, Colombia, and Argentina (other characteristics are in Supplemental Table S2).

### 3.4. Clarity and Comprehension Assessment of the Spanish and Portuguese Versions of the Questionnaire

**Figure 4:**
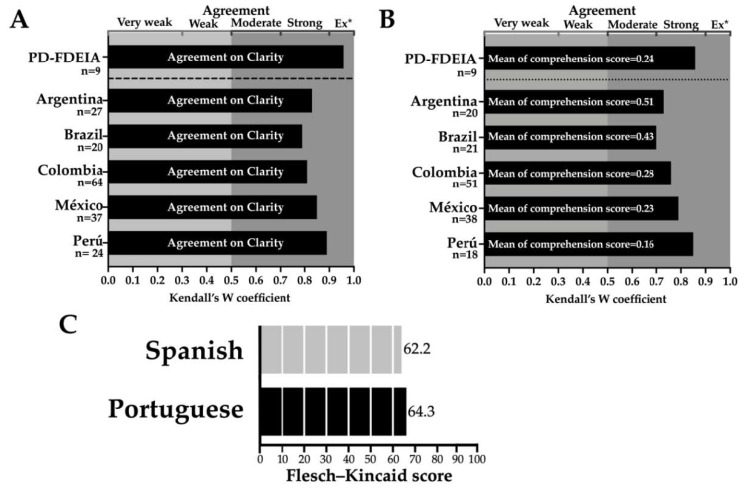
Evaluation of clarity and comprehension. Part (**A**): Evaluation of clarity in a three-point scale (3: clear and comprehensible, 2: difficult to understand, and 1: incomprehensible); Part (**B**): Evaluation of comprehension using a ten-point scale (0 = very easy to understand; 10 = very difficult to understand); Part (**C**): Evaluation of readability using the Flesch–Kincaid score (0 = very complicated to read; 100 = easy to read). A Kendall’s W coefficient of cero (W = 0) means no agreement and W = 1 means total agreement. The Flesch–Kincaid score was calculated by the INFLESZ software. Ex* = Extraordinary agreement; PD-FDEIA: Physician-Diagnosed Food-Dependent Exercise-Induced Anaphylaxis.

According to clarity assessment, the participants classified as clear both the Spanish and Portuguese versions of the questionnaire (Figure 4, part A). For this parameter, concordance among the FDEIA subjects who reported that a physician diagnosed them was extraordinary (Kendall’s W value: 0.958) (Figure 4, part A). Similarly, the degree of agreement among Spanish speakers from different nationalities or Portuguese speakers was strong (Kendall’s W value: 0.81–0.89 and 0.79, respectively) (Figure 4, part A). Participants did not suggest rewording of questions. The comprehension of the questionnaire was assessed using a continuous scale (0: clear and comprehensible, 10: incomprehensible). The averages of the comprehension score ranged from 0.16 to 0.51 among the groups (Figure 4, part B). These values highlight that the questionnaire is “comprehensible”. Furthermore, the agreement scores were “strong” for the two versions of the questionnaire (Kendall’s W values of 0.70 and 0.73–0.85 for the Portuguese and Spanish versions, respectively) (Figure 4, part B). The FDEIA subjects who reported that a physician diagnosed them assigned the average value of 0.24 and the agreement degree was “Strong” (Kendall’s W value of 0.86) (Figure 4, part B). Comprehension scores for each question were ≤3 for >90% of the participants, therefore, rewording of items was not necessary. The Flesch–Kincaid readability scores for the Spanish and Portuguese versions of the questionnaire were 62.2, and 64.3, respectively (Figure 4, part C). This Flesch–Kincaid readability scores are defined as normal (60–70) [26].

### 3.5. Consistency (Repeatability) Assessment

**Figure 5:**
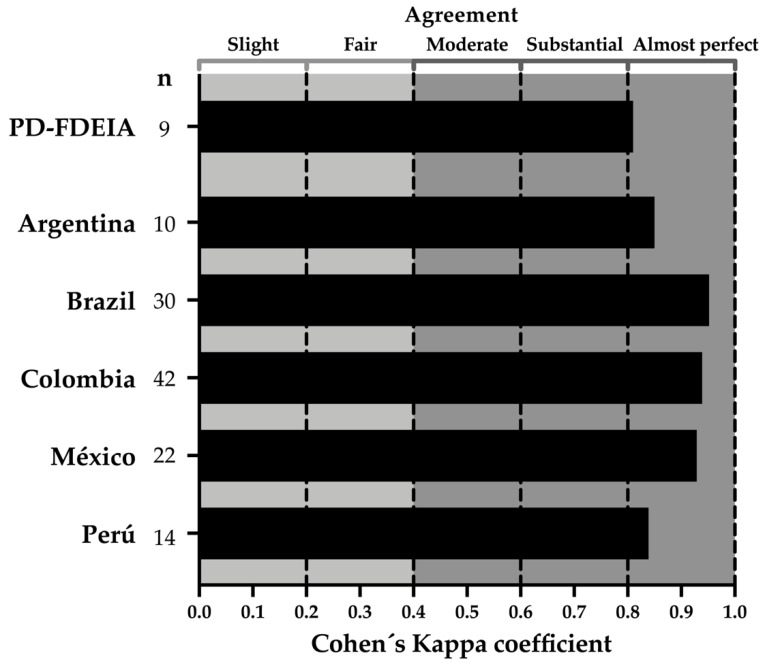
Consistency (repeatability) between responses by the same individual after two applications of the questionnaire. Bars indicate the mean of the Cohen’s Kappa coefficient (degree of coincidences among responses). Dotted lines represent the agreement degree interpretation. The participants answered the questionnaire twice with at least one-week interval between the first and second application. PD-FDEIA: Physician-Diagnosed Food-Dependent Exercise-Induced Anaphylaxis.

The concordance between the responses obtained the first and the second time that the participants answered the questionnaire was assessed employing the Cohen’s k coefficient. The average of Cohen’s k coefficient was 0.81, 0.83–0.93 and 0.95 for the FDEIA subjects who reported that a physician diagnosed them, the Hispanic groups, and the Brazilian group, respectively (Figure 5). These k values can be interpreted as almost perfect concordance among raters. The FDEIA subjects who reported that a physician diagnosed them answered the questionnaire for the first time in an average time of 19.3 min (all questions; mean 19.3; range: 4.43–20.01 min), while the healthy individuals answered it in 2.89 min (three questions; mean 2.89; range: 2.00–4.05 min).

## 4. Discussion

A questionnaire for identifying suspected cases of FDEIA or generating epidemiological data about this condition by self-reporting in Latin American populations was designed and evaluated in the present study. This is the first questionnaire developed for such a purpose. Criteria for making decisions for allocating the participants in one out of three proposed categories were stablished. The proposed categories are self-reported exercise-induced adverse reactions, EIA and FDEIA (either physician-diagnosed or not) and are in agreement with the international definition of anaphylaxis [25]. Some survey-based studies have used this principle of classification and have successfully estimated the self-reported prevalence rates of food-induced anaphylaxis in different Latin American populations [11,12,13]. We took advantage of this knowledge by applying it in a setting were the characteristic symptoms of FDEIA can be triggered. The physical activity options given in the questionnaire were based on the most frequently reported ones by FDEIA cases [19,27]. However, the option “other(s)” was included in order to collect additional information about it. In relation to the intensity of exercise for triggering anaphylaxis, the levels of intensity proposed are in agreement with the American College of Sport Medicine guidelines [21]. Since most FDEIA cases are triggered while or after performing mid intensive physical activity [18], exercise intensity is a parameter of value for identifying potential FDEIA cases. The foods associated with these cases vary from cereals to fruits and vegetables. As options of foods triggering exercise-induced adverse food reactions, the instrument designed in the present study includes the food allergens that according to the codex alimentarius must be declared in prepackaged food products [28]. Foods, such as tomatoes, celery, maize, and peaches have been associated with FDEIA [19,27], but wheat is the most frequently reported food triggering it [3,27,29,30,31]. Overall, the designed instrument encompasses the most important aspects associated with FDEIA, and can be used for identifying potential FDEIA cases or generating epidemiological data about the condition in Latin American countries.

The clarity and comprehension of the Spanish version of the questionnaire was evaluated by native Spanish speakers from four Latin American countries. Clarity and comprehension variables were evaluated in three- and ten-point scales, respectively. Notably, neither the values obtained in the assessments nor the participants per se suggested rewording of questions. Questions rated ≤2 (three-point scale) and >3 (ten-point scale) for ≥10% of the participants indicate that rewording of questions is necessary [16,17]. Similarly, the Flesch–Kincaid score test showed that the instrument developed has a normal complexity to be read for Hispanic people [24]. This test was originally developed to assess texts in English [26] and later adapted for Spanish texts [22]. The number of words by sentence and the number of letters by word are parameters considered for calculating the Flesch–Kincaid score. The k coefficient value obtained in this study (>0.8) can be considered as almost perfect agreement [32], which means that the data obtained with the instrument can be collected in a reproducible manner. Almost perfect Cohen’s k coefficients have been obtained in other studies [16,17]. Further analysis showed that the clarity, comprehension, and repeatability of the instrument developed is also very high when it is applied in the target population. The cohort of FDEIA subjects that participated in the present study seems small, but we should consider that FDEIA is a clinical presentation with a general prevalence ranged from 0.0086 to 0.21% [31]. These results support that the Spanish version of the questionnaire developed is clear and comprehensible enough to be applied in Spanish speaking people from Latin America and that the data generated are reproducible to a large extend.

Due to the Spanish version of the instrument was successfully developed, it was systematically translated to Portuguese, the second most speaking language in South America. The overall match between the two initial translations of the instrument to Portuguese was very high. After the conciliation of conflicting words by two Spanish-Portuguese translators, the conciliated Portuguese version of the questionnaire was back translated to Spanish by two bilingual Spanish/Portuguese (Spanish native speakers). The overall match between the original Spanish version and the two back-translated ones was also very high and the conflicting words were synonymous. This procedure has been evaluated qualitatively [33,34,35,36], but in the present and other studies, we carried out a quantitative evaluation of the translation and back translation procedure through the measurement of the overall match [17]. The high percentage of overall match between the final Spanish version of the questionnaire and the back-translated Portuguese versions of it indicates a very reduced discrepancy [37]. This means that the questions of the Spanish and Portuguese versions of the instrument keep the same meaning. Spanish and Portuguese languages have the highest similarity among romance languages [38], which is advantageous for developing Spanish and Portuguese versions of instruments for carrying out survey studies. Furthermore, the translation back-translation procedure was carried out according to criteria that help to improve the accuracy of the translation [39]. The clarity, comprehension, and repeatability assessment of the Portuguese version of the questionnaire showed scores very similar to, or even higher than, those obtained for the Spanish version. Although, there are no validated tools for evaluating readability in Portuguese, the Flesch-Kincaid readability tests was adapted (Fernandez-Huerta), and this adapted version of the test has allowed to evaluate Portuguese texts [23,40]. Regarding repeatability, the Portuguese version of the questionnaire was almost perfect. The questionnaires validation processes have to ensure that misinterpretations of questions are avoided [14,41]. In the present study, the high W Kendall’s coefficients obtained are the statistical evidence of an almost exact agreement [42] among the perceptions of high clarity and comprehensibility of the questionnaire by individuals from different Latin American countries. Overall, the results of the present study indicate that the questions of the instrument developed are clear, comprehensible and reproducible in both the Spanish and Portuguese versions of it.

## 5. Conclusions

An instrument to identify potential FDEIA cases or generate epidemiological data about this condition by self-report was developed in the present study. The instrument is clear, comprehensible, and generates reproducible data in both Spanish and Brazilian Portuguese languages. The questions and potential responses, given to the interviewees, allow for their classification in one out of five potential categories. As a survey tool, the instrument can be utilized to generate the first epidemiological data about FDEIA in the Latin American region; a region of more than 600 million inhabitants. Mild cultural adaptations of the Spanish or Portuguese version of the questionnaire may be required for applying it in any Latin American country.

## Figures and Tables

**Table 1:** Characteristics of the participants who assessed the questionnaire.

		Clarity	Comprehension	Consistency
Participants (*n*)		181	157	127
Male (*n*)		87	75	58
Female (*n*)		94	82	69
Age in years (range)		18–66	18–66	18–66
Scholarly	Elementary (*n*)	2	2	1
	Junior highschool (*n*)	45	38	28
	Highschool (*n*)	13	12	11
	University (*n*)	99	87	76
	Postgraduate (*n*)	22	18	11

## Supplementary Materials

The following are available online at https://www.mdpi.com/2227-9032/8/4/519/s1, Table S1: Designed questionnaires in Spanish, Portuguese and English. Table S2: FDEIA Cohort.
