# Supplementary material for: Design, Assessment, and Validation of a Questionnaire to Estimate Food-Dependent Exercise-Induced Anaphylaxis Prevalence in Latin American Population
Source: Healthcare (Basel). 2020 Nov 29;8(4):519. doi: 10.3390/healthcare8040519 (PMC7712264; doi:10.3390/healthcare8040519)
Supplement: Supplementary file 1 [file healthcare-08-00519-s001.pdf]

Table S1: Designed questionnaires in spanish, portuguese and english.

Designed questionnaire in spanish

Nombre completo: Edad: años

Sexo: ☐ Femenino ☐ Masculino

1. ¿Le han diagnosticado alguna de las siguientes enfermedades?

Marque solo una opción por fila

a) Asma bronquial	☐ Si	☐ No	☐ No sabe
b) Urticaria	☐ Si	☐ No	☐ No sabe
c) Alergia a la primavera (Rinitis alérgica)	☐ Si	☐ No	☐ No sabe
d) Anafilaxis	☐ Si	☐ No	☐ No sabe
e) Dermatitis atópica	☐ Si	☐ No	☐ No sabe
f) Alergia a insectos	☐ Si	☐ No	☐ No sabe
g) Alergia a los animales	☐ Si	☐ No	☐ No sabe
h) Conjuntivitis alérgica	☐ Si	☐ No	☐ No sabe
i) Alergia a algún medicamento	☐ Si	☐ No	☐ No sabe
J) Enfermedad pulmonar obstructiva crónica (EPOC)	☐ Si	☐ No	☐ No sabe
k) Anafilaxis inducida por el ejercicio	☐ Si	☐ No	☐ No sabe
l) Anafilaxis inducida por el ejercicio dependiente de alimentos	☐ Si	☐ No	☐ No sabe
m) Urticaria colinérgica	☐ Si	☐ No	☐ No sabe
n) Alergia a algún alimento	☐ Si	☐ No	☐ No sabe

2. ¿Ha presentado alguna molestia, síntoma o reacción adversa durante o después de realizar algún tipo de actividad física como caminar, bailar, hacer ejercicio o practicar algún deporte?

Marque solo una opción

☐ Si **(Siga con la pregunta 3)** ☐ No (**Pase a la pregunta número 19**)

3. ¿Cuál de las siguientes actividades practicó o estaba practicando cuando aparecieron los síntomas?

☐ Fútbol	☐ Correr	☐ Subir escaleras	☐ Crossfit
☐ Básquetbol	☐ Trotar	☐ Calistenia	☐ Paseo del perro
☐ Béisbol	☐ Bailar	☐ Limpieza del hogar	☐ Ejercicio de pesas
☐ Caminata	☐ Esquí	☐ Yoga	☐ Ejercicio cardiovascular
☐ Otro ¿cual?_____________			

4. ¿Qué tipo de esfuerzo físico estaba haciendo cuando los síntomas o molestias empezaron?

☐ A) Un esfuerzo que no aceleraba su respiración y le permitía hablar, cantar o silbar con normalidad

☐ B) Un esfuerzo que aceleraba su respiración al punto que se reduce su capacidad para hablar, cantar o silbar

☐ C) Un esfuerzo que aceleraba significativamente su respiración al punto que no podía cantar, hablar o silbar

☐ D) Estaba sentado, acostado descansando (en reposo)

5. ¿Aproximadamente, cuánto tiempo había pasado desde que inicio la actividad física hasta el momento de la aparición de las molestias o síntomas?

Marque solo una opción

☐ Menos de 10 min ☐ 10 min-1 hr ☐ 1-2 hr ☐ 2-4 hr ☐ Más de 4 hr ☐ No sabe

6. ¿Cuál(es) de los siguientes síntomas o molestias presentó cuando realizó la actividad física mencionada anteriormente?

Marque solo una opción por fila

a) Ronchas o sarpullidos que pican	☐ Si	☐ No	☐ No sabe
b) Hinchazón	☐ Si	☐ No	☐ No sabe
¿Dónde? (Puedes marcar mas de una opción)	☐ Si	☐ No	☐ No sabe

☐ Párpados ☐ Labios ☐ Lengua ☐ Cara ☐ Otro: _____________ ☐ No sabe

c) Enrojecimiento de la piel	☐ Si	☐ No	☐ No sabe

¿Dónde? (Puedes marcar mas de una opción)

☐ Brazos/manos ☐ Cuello/cara ☐ Pecho ☐ Todo el cuerpo ☐ Otro ☐ No sabe

Marque solo una opción por fila

d) Vómitos	☐ Si	☐ No	☐ No sabe
e) Dolor de estómago (abdominal)	☐ Si	☐ No	☐ No sabe
f) Diarrea	☐ Si	☐ No	☐ No sabe
g) Náuseas	☐ Si	☐ No	☐ No sabe
h) Congestión nasal	☐ Si	☐ No	☐ No sabe
i) Ojos rojos, picazón ocular, lagrimeo	☐ Si	☐ No	☐ No sabe
j) Rinitis (secreción nasal, lagrimeo ocular y congestión nasal)	☐ Si	☐ No	☐ No sabe
k) Tos	☐ Si	☐ No	☐ No sabe
l) Picazón de garganta	☐ Si	☐ No	☐ No sabe
m) Sensación de garganta apretada	☐ Si	☐ No	☐ No sabe
n) Dificultad para respirar	☐ Si	☐ No	☐ No sabe
o) Silbidos en el pecho	☐ Si	☐ No	☐ No sabe
p) Una baja presión que provoca mareo	☐ Si	☐ No	☐ No sabe
q) Desmayo	☐ Si	☐ No	☐ No sabe

7. ¿Atribuye los síntomas mencionados con anterioridad a que consumió algún alimento y luego realizó alguna actividad física?

Marque solo una opción

☐ Si ☐ No (**Pase a pregunta 12**)

8. ¿Cuánto tiempo paso desde que consumió el alimento y realizó la actividad física que desencadenó los síntomas mencionados anteriormente?

☐ Menos de 10 min ☐ 10 min-1 hr ☐ 1-2 hr ☐ 2-4 hr ☐ Más de 4 hr ☐ No sabe

9. ¿Qué alimento(s) produce(n) las molestias o síntomas mencionados con anterioridad cuando realizó la actividad física?

Puedes marcar una o más de las opciones

☐ Leche	☐ Cacahuate	☐ Nueces	☐ Pescado
☐ Camarón/crustáceos	☐ Soya	☐ Trigo	☐ Huevo
☐ Uvas	☐ Vegetales ¿Cuál?	☐ Otro ¿Cuál?	

10. ¿Los síntomas mencionados con anterioridad se presentan de forma repetida cuando combina la ingesta del alimento con realizar actividad física?

Marque solo una opción

☐ Si ☐ No ☐ No sabe

11. En caso de no realizar actividad física ¿Puede consumir el/los alimento(s) sin presentar algún síntoma o malestar?

Marque solo una opción

☐ Si ☐ No ☐ No sabe

12. ¿Qué edad tenía la primera vez que presentó síntomas o molestias cuando realizó actividad física y había consumido el/los alimento(s) a los que le atribuye el problema? ___________

Marca solo una opción

☐ Menos de 5 años ☐ 5-12 años ☐ 13-18 años ☐ 19-30 años ☐ Otra (indique)_________________

13. ¿Cuántas veces ha presentado los síntomas o molestias ocasionados por la actividad física en combinación con el consumo de el alimento alergénico?

Número de ocasiones ________________ ☐ No sabe

14. ¿Acudió usted a consulta médica por los síntomas que presentó?

Marque solo una opción

☐ Si ☐ No (pase a la pregunta 16) ☐ No sabe (pase a la pregunta 16)

15. ¿Ingresó usted a la sala de urgencias o fue atendido de urgencia por un médico?

Marque solo una opción

☐ Si ☐ No

16. ¿Debió tomar alguno de los siguientes medicamentos debido a los síntomas que presentó?

Puede marcar más de una opción

☐ Antihistamínicos ☐ Adrenalina ☐ Corticoides ☐ Inhaladores ☐ Suero

☐ **No** ☐ **No sabe**

17. ¿Alguna vez se le indicó como parte del tratamiento adrenalina inyectable?

Marque solo una opción

☐ Si ☐ No ☐ No sabe

18. ¿Ha tomado algunas de las siguientes precauciones para evitar tener una nueva reacción durante o después de realizar actividad física?

Puede marcar más de una opción

☐ A) Dejar de consumir alimentos antes o después de realizar actividad física

☐ B) Evitar hacer ejercicio en temporadas de mucho calor o de mucho frío

☐ C) Evitar hacer ejercicio en ambientes húmedos

☐ D) Retirar el alimento por completo de su dieta

☐ E) Cambiar el lugar donde se realiza el ejercicio (en exteriores o interiores)

☐ F) Evitar algunos fármacos como la aspirina o antiinflamatorios

☐ G) Evitar el consumo de alcohol

19. ¿Algún familiar directo (padre, madre o hermanos) presenta alguna de las siguientes enfermedades?

Marque solo una opción por fila

a) Asma bronquial	☐ Si	☐ No	☐ No sabe
b) Alergia alimentaria	☐ Si	☐ No	☐ No sabe
c) Alergia a la primavera (Rinitis alérgica)	☐ Si	☐ No	☐ No sabe
d) Anafilaxis	☐ Si	☐ No	☐ No sabe
e) Dermatitis atópica	☐ Si	☐ No	☐ No sabe
f) Alergia a insectos	☐ Si	☐ No	☐ No sabe
g) Alergia a los animales	☐ Si	☐ No	☐ No sabe
h) Conjuntivitis alérgica	☐ Si	☐ No	☐ No sabe
i) Urticaria	☐ Si	☐ No	☐ No sabe
j) Alergia a algún medicamento	☐ Si	☐ No	☐ No sabe
k) Anafilaxis inducida por el ejercicio	☐ Si	☐ No	☐ No sabe
l) Anafilaxis inducida por el ejercicio dependiente de alimentos	☐ Si	☐ No	☐ No sabe
m) Urticaria colinérgica	☐ Si	☐ No	☐ No sabe

n) Alergia a algún alimento	☐ Si	☐ No	☐ No sabe

Designed questionnaire in portuguesse

Nome completo: Idade: anos

Sexo: ☐ Feminino ☐ Masculino

1. Você já foi diagnosticado com alguma das seguintes doenças?

Marque uma única opção por fila

a) Asma brônquica	☐ Sim	☐ Não	☐ Não sabe
b) Urticaria	☐ Sim	☐ Não	☐ Não sabe
c) Alergia de primavera (Rinite alérgica)	☐ Sim	☐ Não	☐ Não sabe
d) Anafilaxia	☐ Sim	☐ Não	☐ Não sabe
e) Dermatite atópica	☐ Sim	☐ Não	☐ Não sabe
f) Alergia a insetos	☐ Sim	☐ Não	☐ Não sabe
g) Alergia a animais	☐ Sim	☐ Não	☐ Não sabe
h) Conjuntivite alérgica	☐ Sim	☐ Não	☐ Não sabe
i) Alergia a algum medicamento	☐ Sim	☐ Não	☐ Não sabe
J) Doença Pulmonar Obstrutiva Crônica (DPOC)	☐ Sim	☐ Não	☐ Não sabe
k) Anafilaxia induzida por exercício	☐ Sim	☐ Não	☐ Não sabe
l) Anafilaxia induzida por exercício dependente de alimentos	☐ Sim	☐ Não	☐ Não sabe
m) Urticaria colinérgica	☐ Sim	☐ Não	☐ Não sabe
n) Alergia a algum alimento	☐ Sim	☐ Não	☐ Não sabe

2. Você tem apresentado algum desconforto, sintoma ou reação adversa durante ou depois de realizar algum tipo de atividade física como caminhar, dançar, fazer exercício o praticar algum esporte?

Marque apenas uma opção

☐ Sim **(Siga com a pergunta 3)** ☐ Não (**Passe a pergunta número 19**)

3. Qual das seguintes atividades você praticou ou estava praticando quando apareceram os sintomas?

☐ Futebol	☐ Correr	☐ Subir escadas	☐ Crossfit
☐ Basquete	☐ Trotar	☐ Calistenia	☐ Passeio com o cachorro
☐ Beisebol	☐ Dançar	☐ Limpeza da casa	☐ Academia (exercícios com pesos)
☐ Caminhada	☐ Esqui	☐ Yoga	☐ Academia (exercícios cardiovasculares)
☐ Outro ¿Qual?			

4. Que tipo de esforço físico você estava fazendo quando os sintomas ou desconforto começaram?

☐ A) Um esforço que não acelerava sua respiração e permitia falar, cantar ou assobiar com normalidade

☐ B) Um esforço que acelerava sua respiração ao ponto que reduz sua capacidade para falar, cantar ou assobiar

☐ C) Um esforço que acelerava significativamente sua respiração ao ponto que não podia cantar, falar ou assobiar

☐ D) Estava sentado, deitado descansando (em repouso)

5. Aproximadamente, quanto tempo havia passado desde que iniciou a atividade física até o momento da aparição do desconforto ou sintomas?

Marque apenas uma opção

☐Menos de 10 min ☐ 10 min-1 hr ☐ 1-2 hr ☐ 2-4 hr ☐ Mais de 4 hr ☐ Não sabe

6. Qual(is) dos seguintes sintomas ou desconforto você apresentou quando realizou a atividade física mencionada anteriormente?

Marque apenas uma opção por fila

a) Urticária ou erupções que coceira ☐Sim ☐ Não ☐ Não sabe

b) Inchaço ☐Sim ☐ Não ☐ Não sabe

Onde? (Você pode marcar mais de uma opção)

☐Pálpebras ☐ Lábios ☐ Língua ☐ Cara ☐ Outro:_______________ ☐Não sabe

c) Vermelhidão da pele ☐ Sim ☐ Não ☐ Não sabe

Onde? (Você pode marcar mais de uma opção)

☐ Braços/mãos ☐ Pescoço/cara ☐ Peito ☐ Todo o corpo ☐ Outro ☐Não sabe

Marque apenas uma opção por fila

d) Vômitos ☐ Sim ☐ Não ☐ Não sabe

e) Dor de estômago (abdominal) ☐ Sim ☐ Não ☐ Não sabe

f) Diarreia ☐ Sim ☐ Não ☐ Não sabe

g) Náuseas ☐ Sim ☐ Não ☐ Não sabe

h) Congestão nasal ☐ Sim ☐ Não ☐ Não sabe

i) Olhos vermelhos, olho com coceira, lacrimejo ☐ Sim ☐ Não ☐ Não sabe

j) Rinites (secreção nasal, lacrimejo ocular e congestão nasal) ☐ Sim ☐ Não ☐ Não sabe

k) Tosse ☐ Sim ☐ Não ☐ Não sabe

l) Coceira na garganta ☐ Sim ☐ Não ☐ Não sabe

m) Sensação de aperto na garganta ☐ Sim ☐ Não ☐ Não sabe

n) Dificuldade para respirar ☐ Sim ☐ Não ☐ Não sabe

o) Assbios no peito ☐ Sim ☐ Não ☐ Não sabe

p) Uma baixa pressão que provoca tontura ☐ Sim ☐ Não ☐ Não sabe

q) Desmaio ☐ Sim ☐ Não ☐ Não sabe

7. Você atribui os sintomas mencionados anteriormente a algum alimento que consumiu e logo realizou alguma atividade física?

Marque apenas uma opção por fila

Sim Não (**Passe a pergunta 12**)

8. Quanto tempo passou desde que você consumiu o alimento e realizou a atividade física que desencadeou os sintomas mencionados anteriormente?

☐Menos de 10 min ☐ 10 min-1 hr ☐ 1-2 hr ☐ 2-4 hr ☐ Mais de 4 hr ☐ Não sabe

9. Que alimento(s) produz(em) o desconforto ou sintomas mencionados anteriormente quando realizou a atividade física?

Pode marcar uma ou mais opções

☐ Leite ☐ Amendoim ☐ Nozes ☐ Peixe

☐ Camarão/crustáceos ☐ Soja ☐ Trigo ☐ Ovo

☐ Uvas ☐ Vegetais, Qual? ☐ Outro, Qual?

10. Os sintomas mencionados anteriormente se apresentam de forma repetida quando combina a ingestão de alimento com realizar atividade física?

Marque apenas uma opção

☐ Sim ☐ Não ☐ Não sabe

11. No caso de não realizar atividade física, você pode consumir o/os alimento(s) sem apresentar algum sintoma ou mal-estar?

Marque apenas uma opção

☐ Sim ☐ Não ☐ Não sabe

12. Que idade você tinha a primeira vez que apresentou os sintomas ou desconfortos quando realizou atividade física e havia consumido o/os alimento(s) que você atribui o problema? ___________

Marque apenas uma opção

☐ Menos de 5 anos ☐ 5-12 anos ☐ 13-18 anos ☐ 19-30 anos ☐ Outra (indique)________________

13. Quantas vezes você já teve sintomas ou desconfortos causados por atividades físicas quando combina a ingestão de alimento alergênico?

Número de vezes ________________ ☐ Não sabe

14. Você foi a uma consulta médica pelos sintomas que apresentou?

Marque apenas uma opção

☐ Sim ☐ Não (passe a pergunta 16) ☐ Não sabe (passe a pergunta 16)

15. Você foi ao pronto-socorro ou foi atendido com urgência por um médico??

Marque apenas uma opção

☐ Sim ☐ Não

16. Você tomou algum dos seguintes medicamentos devido aos sintomas que apresentou?

Pode marcar mais de uma opção

☐ Anti-histamínicos ☐ Adrenalina ☐ Corticoides ☐ Inaladores ☐ Soro

☐ **Não** ☐ **Não sabe**

17. Você já recebeu injeção de adrenalina como parte do tratamento?

Marque apenas uma opção

☐ Sim ☐ Não ☐ Não sabe

18. Você tem tomado algumas das seguintes precauções para evitar uma nova reação durante ou depois de realizar a atividade física?

Pode marcar mais de uma opção

☐ A) Deixar de consumir alimentos antes ou depois de realizar atividade física

☐ B) Evitar fazer exercício em temporadas de muito calor ou de muito frio

☐ C) Evitar fazer exercício em ambientes úmidos

☐ D) Retirar o alimento por completo da sua dieta

☐ E) Mudar o lugar onde se realiza o exercício (ao ar livre ou dentro de casa)

☐ F) Evitar alguns medicamentos como a aspirina ou anti-inflamatórios

☐ G) Evitar o consumo de álcool

19. Algum familiar direto (pai, mãe ou irmãos) apresenta alguma das seguintes doenças?

	**Marque uma única opção por fila**		
a) Asma brônquica	☐ Sim	☐ Não	☐ Não sabe
b) Urticaria	☐ Sim	☐ Não	☐ Não sabe
c) Alergia de primavera (Rinite alérgica)	☐ Sim	☐ Não	☐ Não sabe
d) Anafilaxia	☐ Sim	☐ Não	☐ Não sabe
e) Dermatite atópica	☐ Sim	☐ Não	☐ Não sabe
f) Alergia a insetos	☐ Sim	☐ Não	☐ Não sabe
g) Alergia a animais	☐ Sim	☐ Não	☐ Não sabe
h) Conjuntivite alérgica	☐ Sim	☐ Não	☐ Não sabe
i) Alergia a algum medicamento	☐ Sim	☐ Não	☐ Não sabe
J) Doença Pulmonar Obstrutiva Crônica (DPOC)	☐ Sim	☐ Não	☐ Não sabe
k) Anafilaxia induzida por exercício	☐ Sim	☐ Não	☐ Não sabe
l) Anafilaxia induzida por exercício dependente de alimentos	☐ Sim	☐ Não	☐ Não sabe
m) Urticaria colinérgica	☐ Sim	☐ Não	☐ Não sabe
n) Alergia a algum alimento	☐ Sim	☐ Não	☐ Não sabe

Designed questionnaire in English (not evaluated in the present study)

Name: age: _______years old

Sex: ☐Female ☐Male

1. Have you been diagnosed with any of the following diseases?

Select only one option per row

a) Bronchial asthma	☐ Yes	☐ No	☐ I do not know
b) Urticaria	☐ Yes	☐ No	☐ I do not know
c) Spring allergy (Allergic rhinitis)	☐ Yes	☐ No	☐ I do not know
d) Anaphylaxis	☐ Yes	☐ No	☐ I do not know
e) Atopic dermatitis	☐ Yes	☐ No	☐ I do not know
f) Insect allergy	☐ Yes	☐ No	☐ I do not know
g) Animal allergy	☐ Yes	☐ No	☐ I do not know
h) Allergic conjunctivitis	☐ Yes	☐ No	☐ I do not know
i) Drug allergy	☐ Yes	☐ No	☐ I do not know
J) Chronic obstructive pulmonary disease (COPD)	☐ Yes	☐ No	☐ I do not know
k) Exercise-induced anaphylaxis	☐ Yes	☐ No	☐ I do not know
l) Food-dependent exercise-induced anaphylaxis	☐ Yes	☐ No	☐ I do not know
m) Cholinergic urticaria	☐ Yes	☐ No	☐ I do not know
n) Food Allergy	☐ Yes	☐ No	☐ I do not know

2. Have you experienced any discomfort, symptom or adverse reaction while or after performing some kind of physical activity such as walking, dancing, exercising or playing sports?

Select only one option

☐ Yes **(Go to question 3)** ☐ No (**Go to question number 19**)

3. Which of the following activities were you performing when the symptoms appeared?

☐ Soccer	☐ Run	☐ Climbing stairs	☐ Crossfit
☐ Basketball	☐ Jogging	☐ Climbing stairs	☐ Walking the dog
☐ Baseball	☐ Dance	☐ Household cleaning	☐ Weight exercise
☐ Walk	☐ Ski	☐ Yoga	☐ Cardio

☐ Other, Which one?____________

4. What kind of physical effort were you doing when the symptoms or discomfort started?

☐ A) An effort that did not speed up his breathing and allowed him to speak, sing or whistle normally.

☐ B) An effort that sped up your breathing to the point that it reduced your ability to speak, sing, or whistle.

☐ C) An effort that significantly accelerated his breathing to the point that he could not sing, speak or whistle.

☐ D) I was sitting, lying resting (resting)

5. Approximately, how long had it been since you started physical activity until the discomfort or symptoms appeared?

Select only one option

☐ Less than 10 min ☐ 10 min-1 hrs. ☐ 1-2 hrs. ☐ 2-4 hrs. ☐ More than 4 hrs. ☐ I do not know

6. Which of the following symptoms or discomforts did you experience while performing the physical activity mentioned above?

Select only one option per row

a) Sink with hives	☐ Yes	☐ No	☐ I do not know
b) Swelling	☐ Yes	☐ No	☐ I do not know
¿Where? (You can mark more than one option)	☐ Yes	☐ No	☐ I do not know

☐ Eyelids ☐ Lips ☐ Tongue ☐ Face ☐ Other: _____________ ☐ I do not know

c) Skin redness	☐ Yes	☐ No	☐ I do not know

¿Where? (You can mark more than one option)

☐ Arms/hands ☐ Neck/face ☐ Chest ☐ The whole body ☐ Other ☐ I do not know

Select only one option per row

d) Vomiting	☐ Yes	☐ No	☐ I do not know
e) Abdominal pain	☐ Yes	☐ No	☐ I do not know
f) Diarrhea	☐ Yes	☐ No	☐ I do not know
g) Ssickness	☐ Yes	☐ No	☐ I do not know
h) Nasal congestion	☐ Yes	☐ No	☐ I do not know
i) Red eyes, eye itching, tearing	☐ Yes	☐ No	☐ I do not know
j) Rhinitis	☐ Yes	☐ No	☐ I do not know
k) Cough	☐ Yes	☐ No	☐ I do not know
l) Itchy throat	☐ Yes	☐ No	☐ I do not know
m) Throat tightness	☐ Yes	☐ No	☐ I do not know
n) Trouble breathing	☐ Yes	☐ No	☐ I do not know
o) Wheezing	☐ Yes	☐ No	☐ I do not know
p) Low pressure	☐ Yes	☐ No	☐ I do not know
q) Fainting	☐ Yes	☐ No	☐ I do not know

7. Do you attribute the symptoms mentioned above to eating some food and then doing some physical activity?

Select only one option

☐ Yes ☐ No (**Go to question 12**)

8. How long did it took since you ate the food and performed the physical activity that triggered the symptoms mentioned above?

☐ Less than 10 min ☐ 10 min-1 hrs. ☐ 1-2 hrs. ☐ 2-4 hrs. ☐ More than 4 hrs. ☐ I do not know

9. What food(s) causes the aforementioned discomfort or symptoms when performing physical activity?

You can mark one or more of the options

☐ Milk ☐ Peanut ☐ Nuts ☐ Fish

☐ Shrimp/crustaceans ☐ Soy ☐ Wheat ☐ Egg

☐ Grapes ☐ Vegetables, which one? ☐ Other, which one?

10. Do the symptoms mentioned above occur repeatedly when you combine food intake with physical activity?

Select only one option

☐ Yes ☐ No ☐ I do not know

11. If you are not physically active, can you eat the food(s) without experiencing any symptom or discomfort?

Select only one option

☐ Yes ☐ No ☐ I do not know

12. How old were you the first time that you experienced symptoms or discomfort while or after you were performing physically activity and ate the foods you attribute the problem? ___________

Select only one option

☐ Less than 5 years old ☐ 5-12 years old ☐ 13-18 years old ☐ 19-30 years old ☐ Other (please provide)_________

13. How many times have you had symptoms or discomfort caused by physical activity in combination with the consumption of the allergenic food?

Number of times________________ ☐ I do not know

14. Did you go to the doctor for your symptoms?

Select only one option

☐ Yes ☐ No (Go to question 16) ☐ I do not know (Go to question 16)

15. Did you go to the emergency room or were you treated urgently by a doctor?

Select only one option

☐ Yes ☐ No

16. Have you taken any of the following medications because of your symptoms?

You can select more than one option

☐ Antihistamines ☐ Adrenaline ☐ Corticosteroids ☐ Inhalers ☐ Serum

☐ **No** ☐ **I do not know**

17. Have you ever had an adrenaline injection as part of treatment?

Select only one option

☐ Yes ☐ No ☐ I do not know

18. Have you taken any of the following precautions to prevent a new reaction while or after performing physical activity?

You can select more than one option

☐ A) Stop consuming food before or after physical activity

☐ B) Avoid exercising in very hot or cold seasons.

☐ C) Avoid exercising in humid environments.

☐ D) Completely eliminate foods from your diet.

☐ E) Change the place where the exercise is carried out (outside or inside)

☐ F) Avoid some medications such as aspirin or anti-inflammatories.

☐ G) Avoid drinking alcohol

☐ H) None

19. Does any of your first-degree relatives (father, mother or siblings) have any of the following diseases?

Select only one option per row

a) Bronchial asthma	☐ Yes	☐ No	☐ I do not know
b) Urticaria	☐ Yes	☐ No	☐ I do not know
c) Spring allergy (Allergic rhinitis)	☐ Yes	☐ No	☐ I do not know
d) Anaphylaxis	☐ Yes	☐ No	☐ I do not know
e) Atopic dermatitis	☐ Yes	☐ No	☐ I do not know
f) Insect allergy	☐ Yes	☐ No	☐ I do not know
g) Animal allergy	☐ Yes	☐ No	☐ I do not know
h) Allergic conjunctivitis	☐ Yes	☐ No	☐ I do not know
i) Drug allergy	☐ Yes	☐ No	☐ I do not know
J) Chronic obstructive pulmonary disease (COPD)	☐ Yes	☐ No	☐ I do not know
k) Exercise-induced anaphylaxis	☐ Yes	☐ No	☐ I do not know

l) Food-dependent exercise-induced anaphylaxis	☐ Yes	☐ No	☐ I do not know
m) Cholinergic urticaria	☐ Yes	☐ No	☐ I do not know
n) Food Allergy	☐ Yes	☐ No	☐ I do not know

Table S2. FDEIA Cohort

	Characteristics	n
**Sex**	Male	2
	Female	7
**Type of physical activity**	Soccer	3
	Gym	3
	Running	2
	Basketaball	1
**Intensity of physical activity**	Vigorous	6
	Moderate	2
	Rest	1
**Foods**	Milk and wheat	2
	Shrimp	2
	Milk and nuts	1
	Milk	1
	Nuts	1
	Not reported	1
**Precautions to prevent a new episode**	Avoided some medications such as aspirin or anti-inflammatory drugs	
	Stopped consuming the suspicious food before or after physical activity	2
	Did not take any precautions	2
	Subject avoided exercising in hot seasons or very cold	
	Avoid exercising in humid environments	1
	Change the place where the exercise is performed (outside or inside), avoid alcohol consumption	
	Subject completely eliminated the suspicious food from her diet	1
	Stopped consuming food before or after physical activity	1
